# Graphene Oxide Induced Osteogenesis Quantification by In-Situ 2D-Fluorescence Spectroscopy

**DOI:** 10.3390/ijms19113336

**Published:** 2018-10-26

**Authors:** Valentina Palmieri, Marta Barba, Lorena Di Pietro, Claudio Conti, Marco De Spirito, Wanda Lattanzi, Massimiliano Papi

**Affiliations:** 1 Institute of Physics, Fondazione Policlinico Universitario A. Gemelli-IRCCS, Università Cattolica del Sacro Cuore, Largo Francesco Vito 1, 00168 Rome, Italy; valentina.palmieri@unicatt.it (V.P.); marco.despirito@unicatt.it (M.D.S.); 2 Institute for Complex Systems, National Research Council (ISC-CNR), Via dei Taurini 19, 00185 Rome, Italy; claudioconti@uniroma1.it; 3 Institute of Anatomy and Cell Biology, Fondazione Policlinico Universitario A. Gemelli-IRCCS, Università Cattolica del Sacro Cuore, Largo Francesco Vito 1, 00168 Rome, Italy; marta.barba@unicatt.it (M.B.); lorena.dipietro@unicatt.it (L.D.P.); wanda.lattanzi@unicatt.it (W.L.)

**Keywords:** Alizarin Red S, osteogenesis, fluorescence spectroscopy, graphene oxide

## Abstract

Graphene and graphene oxide can promote the adhesion, growth and differentiation of mesenchymal stem cells. Further, graphene surface coatings accelerate the differentiation of human mesenchymal stem cells acting as osteogenic inducers. Quantification of the osteogenic induction is conventionally performed with Alizarin Red S (ARS), an anthraquinone derivative used to identify calcium deposits in tissue sections and cell cultures. The ARS staining is quite versatile because the dye forms an Alizarin Red S–calcium complex that can be extracted from the stained monolayer of cells and readily assayed by absorbance measurements. Direct visualization of stained deposits is also feasible; however, an in-situ visualization and quantification of deposits is possible only on transparent supports and not on thick opaque materials like ceramics and graphene composites that are well-known inducers of osteogenesis. In this manuscript, the shape of the 2D-fluorescence spectra of the ARS-calcium complex is used to develop a method to detect and monitor the in-situ differentiation process occurring during the osteogenic induction mediated by opaque graphene oxide surfaces.

## 1. Introduction

Bone engineering is aimed at bone repair and regeneration with materials able to restore original tissue properties. In the last ten years, graphene, graphene oxide (GO) and their composites have emerged as important game changers in this field. Indeed, by forming coatings and scaffolds made of these bi-dimensional carbon allotropes, it has been demonstrated that graphene-based material (GBM) can favor the differentiation and growth of mesenchymal stem cells and ultimately induce bone formation [1]. The process of osteogenic induction seems to be driven and modulable by the peculiar surface chemistry and hydrophobicity of GO [1,2]. In addition to this, GBMs have excellent tunable mechanical properties and antibacterial properties and have been extensively studied as a future prosthetic material.

The osteogenic processes occurring on scaffold and coating materials are usually quantified with Alizarin Red S (ARS) staining. This is the standard method used to evaluate calcium-rich deposits produced by cells of the osteogenic lineage in culture, and is extremely useful for testing matrix mineralization induced by osteo-inductive treatments [3]. ARS staining can be used for a qualitative measurement of calcification with optical microscopy or to quantify the amount of deposits with spectroscopy techniques [3]. This method quantifies osteogenesis after solvent extraction of the stained deposits from the material surface [3]. In situ visualization of deposits can be obtained after ARS staining only on transparent materials, such as glass slides or cell culture plastic ware.

GBM and most of the materials used for implants, are however, opaque or have irregular features capable of influencing absorbance quantification [4]. A method to quantify ARS in situ could be extremely useful to assess the matrix distribution on complex and opaque surfaces, such as ceramics and titanium implants, graphene and metal alloys, as well as materials with geometrical patterns or interfaces between different composites [5]. In situ quantification would avoid debris loss by fragile material surfaces after solvent extraction, and would be suitable for in vivo experimental procedures.

It is known that ARS exhibits fluorescence in mineralized bone after UV excitation. This fluorescence signal has been used for qualitative in vivo imaging [6,7]. In this paper, we analyzed ARS fluorescence using 2D spectra. Because the 2D spectra shape exhibits specific modifications during ARS–calcium complex formation, we propose a method for in situ quantification of osteogenic induction on Graphene Oxide (GO), which is also feasible for other opaque materials. This method is based on the ratio between the red fluorescence emission peak (670 nm) and the saddle point at 560 nm in order to quantify the osteogenesis induction without ARS extraction from the surface. This method has comparable results to absorbance measurements but allows a fast topographical quantification of bone mineralization via spectroscopy, avoids artifacts due to its extraction procedures, and is feasible on any kind of material used for bone engineering.

## 2. Results

The production of extracellular calcium deposits by calvarial-derived mesenchimal stem cells (CMSCs), a process called mineralization, is an indication of successful in vitro bone formation and can specifically be followed by using ARS [8].

**Figure 1:**
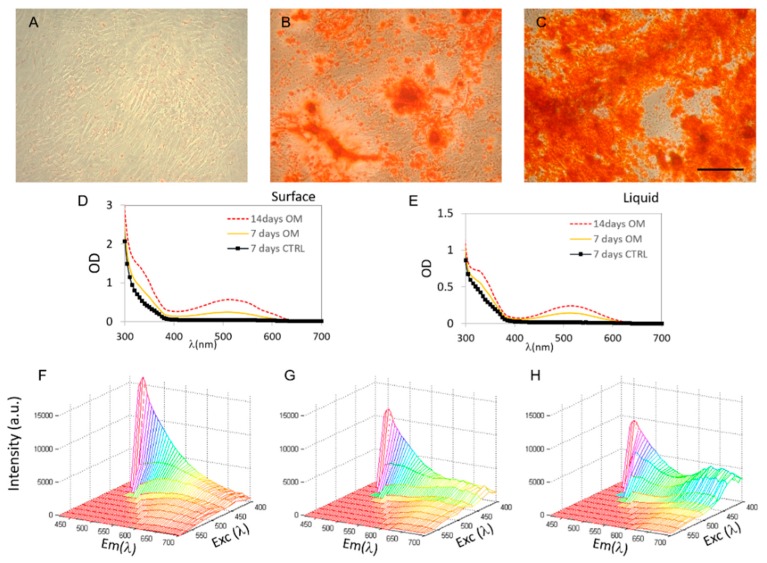
Alizarin Red S (ARS) staining of calvarial-derived mesenchimal stem cells (CMSC) on transparent surfaces. CMSC after 7 days (**A**), 7 days after OM induction (**B**), 14 days after osteogenic medium (OM) induction (**C**). The scale bar for (**A**–**C**) is reported in (**C**) and the value is 50 µm. Adsorption spectra of samples obtained in situ (**D**) or after solvent extraction (**E**). Fluorescence 2D spectra are reported in (**F**–**H**) for 7 days, 7 days after OM induction, and 14 days after OM induction, respectively.

In Figure 1, three representative optical microscopy images of CMSCs cells grown on glass coverslips and stained with ARS are shown. A clear induction of osteogenesis with respect to control cells (Figure 1A), is visible in the presence of osteogenic medium (OM) after seven days (Figure 1B) and 14 days (Figure 1C).

Matrix mineralization is visible from the appearance of red extracellular deposits and nodule formation (Figure 1B,C). The adsorption spectra of samples grown on glass are reported in Figure 1D (measured directly on surface) and 1e (measured after solvent extraction) and display the typical peaks of ARS [3]. The optical density (OD) peak at 515 nm is sensitive to osteogenesis and can be used to quantify the extent of the matrix production both locally (i.e., directly on the surface) or after extraction of the calcified mineral from the stained monolayer. However, OD is not suitable to directly measure the morphological distribution of ARS stained deposits on opaque materials. For this reason, we characterized ARS fluorescence excitation and emission directly on the material surface in the control sample after seven days (Figure 1F), and on the sample induced with OM after 7 and 14 days, in Figure 1G and Figure 1H respectively. These 2D spectra result from wavelengths of excitation (*λ* EXC) of the sample from 400 nm up to 580 nm, and from emissions recorded from *λ* EXC + 40 nm up to 700 nm.

The spectra display a tridimensional shape that varies as osteogenesis moves forward: from a single 500 nm peak in undifferentiated cells, to a double-peaked curve, with a peak at 670 nm appearing in cells treated with osteo-inductive treatment. A local minimum in a saddle point is visible at an emission wavelength of 560 nm.

**Figure 2:**
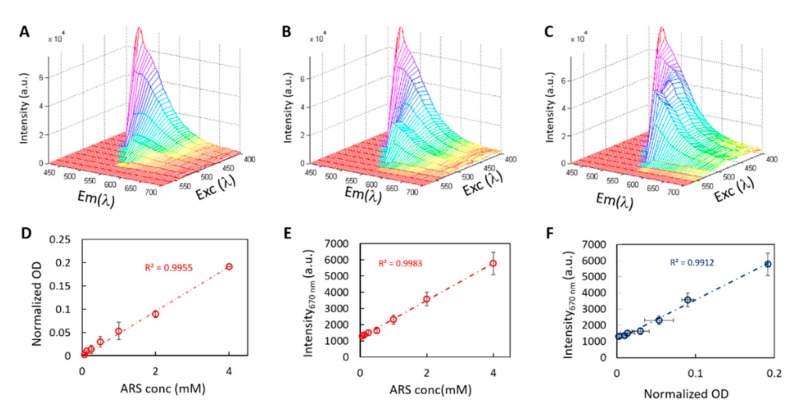
2D spectra of ARS at increasing concentration: 0.25 mM (**A**), 1 mM (**B**), 4 mM (**C**). OD peak vs. concentration is reported in (**D**) while fluorescence peak at 670 nm (excitation 420 nm) is in (**E**). In (**F**), quantification with the two different methods is plotted. Dashed lines represent the linear fit with the corresponding *R*^2^ value shown.

This characteristic variation of the fluorescence is specifically related to the differentiation process, in which the contribution of the cellular fluorescence decreases in favor of the extracellular matrix production, hence the formation of the ARS–calcium complex (peak at 670 nm). Indeed, by measuring the spectrum of ARS staining alone in solution at different concentrations, the variation of the 500 nm peak is not visible and there is no saddle point formation. A rigid linear shift of the spectra intensity with concentration is visible, without any significant change in the spectra shape (Figure 2A: ARS at a 0.25 mM concentration; Figure 2B: 1 mM concentration and; Figure 2C: 4 mM concentration).

In Figure 2D,E, OD and fluorescence intensity peaks at 670 nm (*λ* EXC = 420 nm) of the ARS molecule are shown in relation to the ARS staining concentration. Both display a linear relationship with *R*^2^ > 0.99 (Figure 2D for the OD and Figure 2E for the fluorescence peak at 670 nm exciting at 420 nm). In Figure 2F, the increase in the OD has been related to an increase in the fluorescence peak and a linear fit is shown with *R*^2^ = 0.99.

The shape modification of the fluorescence 2D spectrum and the typical saddle point formation, require a sensitive fingerprint to directly visualize the process of osteogenesis by means of the ARS–Calcium complex formation. Furthermore, the relative increase of the 670 nm peak could be used, similarly to the OD, as an optical method to quantify the ARS concentration, with the advantages of in-situ applicability on opaque materials. To achieve an easy quantification of the relative fluorescence shape modification, we calculated a normalized value defined as the ratio between the intensity value of the emission at the local maximum at 670 nm, and the intensity value at the saddle point at 560 nm (measured with *λ* EXC) of 420 nm.

**Figure 3:**
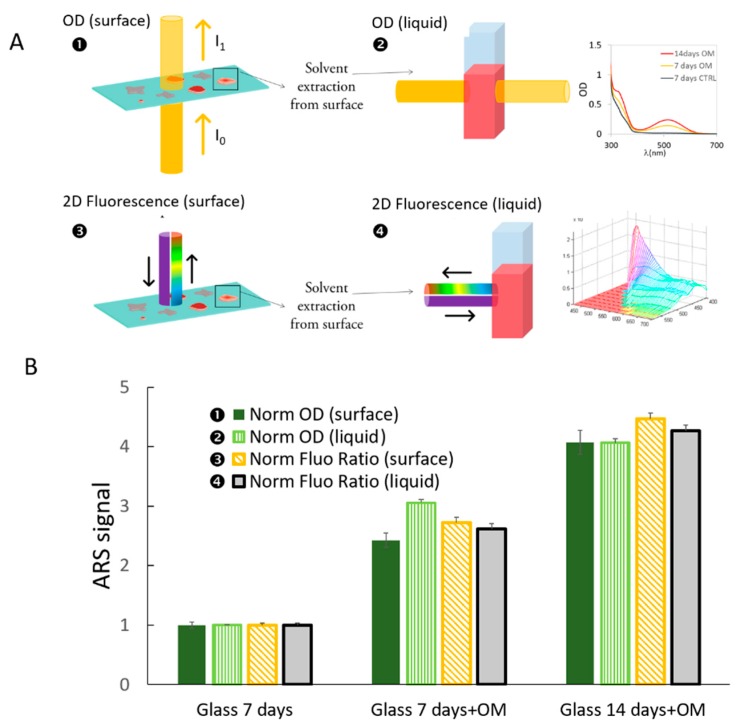
(**A**) Illustration of the four methods used for quantification of ARS staining. The OD and Normalized fluorescence ratios were both quantified after solvent extraction (“liquid”) or directly in situ (“surface”). (**B**) Histogram of methods used to quantify mineralization via ARS staining. Three samples are compared: glass after seven days without induction, and glass after 7 and 14 days with OM induction.

In Figure 3, we compare four different methods to quantify the osteogenic process: two methods based on the OD, i.e., in situ and after solvent extraction, and the two methods proposed here based on the fluorescence-normalized index, again in situ or after solvent extraction (see illustration on methods, Figure 3A). Quantification was performed for cells grown on glass coverslips and with differentiation induced after 7 and 14 days by the OM. The data were normalized to the control sample (cells on glass without induction). Based on the value reported in the histograms, the four different optical methods can quantify cell differentiation with a numerical difference within 10%.

**Figure 4:**
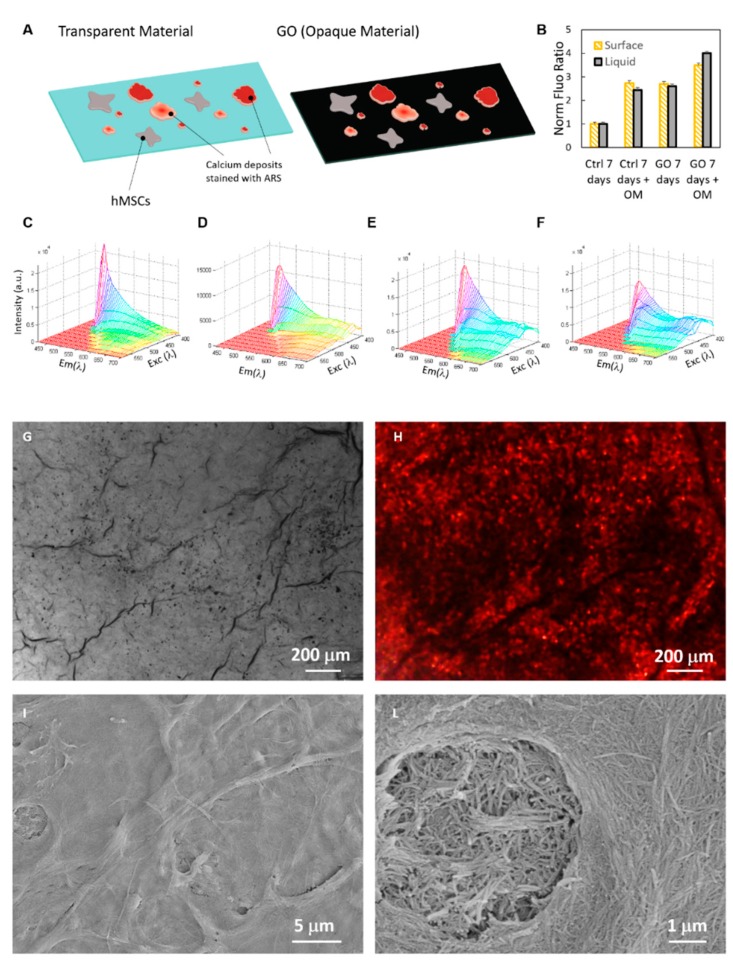
ARS quantification on graphene oxide (GO) substrates. As is visible from the schematic illustrations, we compared the quantification of calcium deposits on transparent substrates, i.e., glass, and on opaque materials i.e., GO (**A**). Results of fluorescence analysis of different samples are reported in histogram (**B**). 2D spectra of samples on glass after seven days (**C**), on glass seven days after OM induction (**D**), on GO after seven days (**E**) and on GO seven days after OM induction (**F**). Representative bone matrix on the GO surface imaged with optical microscopy (**G**), fluorescence (**H**) and scanning electron microscopy (**I**,**L**), respectively.

To test the application of this optical method on opaque and absorbing light surfaces, we prepared substrates of GO, a very promising material used for medical device coating and scaffolds for tissue engineering [1,9,10,11,12,13] and a well-known inducer of osteogenesis [14] and light adsorbing material [15]. Cells and calcium deposits grown on GO are not visible in standard transmission optical microscopy because of the opacity of this material and the in situ osteogenic quantification by OD is not possible due to the light adsorption from GO (Figure 4A). By means of the 2D-fluorescence spectra shape, we were able to identify the osteogenic process of CMSCs by the saddle point formation and to quantify the in-situ ARS–calcium complex formation by using the normalized index (as summarized in Figure 4B–F). After seven days, the 2D fluorescence shape clearly revealed the auto-differentiation triggered by the GO surface itself (Figure 4E), whereas by treating cells with the OM, the spectra manifest a broader shape modification. The quantification of osteogenic induction by GO substrate is shown in Figure 4B. Results after seven days and seven days + OM are plotted in the histogram and compared to the control sample. The osteogenesis is caused by the GO surface without growth factors and is, as expected, enhanced by the OM when cells are grown on GO material, with a fold induction of ~2.7 and ~3.5 to control sample, respectively. These values agree with those reported in the literature [2]. Finally, because we could not compare these results with the standard optical OD techniques, we checked the effective formation of an extracellular matrix on the GO surface during the saddle point formation without OM treatment. GO samples were observed by fluorescence microscopy (Figure 4G,H) and scanning electron microscopy (Figure 4I,L). High-resolution microscopy revealed the presence of extracellular structures as soon as the fluorescence spectra exhibited a shape modification.

## 3. Discussion

Researchers in the field of tissue engineering are investigating new techniques for the regeneration and repair of lost and damaged tissues, and for an accurate visualization/quantification of the restored tissues both in vitro and in vivo.

Among several other advantages, including excellent mechanical properties and good biocompatibility, graphene was demonstrated to be effective in directing stem cell differentiation. It was originally demonstrated in 2010 that graphene can promote the adhesion and growth of mesenchymal stem cells and osteogenic differentiation [16]. Since this pioneering work, others have proved graphene concentration of osteogenic precursors, the possibility to spatially control calcium deposit distribution on laser reduced GO, and the successful production of GO-based scaffolds for bone regeneration [1,17,18].

The osteogenic process is conventionally quantified with Alizarin Red S staining.

ARS is a well-established method to characterize a mineralized matrix due to the differentiation of osteogenic lineage cells, such as CMSCs. Even though a variety of optical biosensors that undergo conformational changes in fluorescent emission upon binding calcium, have been developed over recent years [19,20], Alizarin Red staining continues to hold many advantages [6]. ARS staining allows the simultaneous evaluation of mineral distribution and inspection of structures by optical microscopy and ARS dye can be extracted from the stained monolayer and quantified [3].

However, since GO is an opaque material, an accurate in situ quantification of the mineralization process is challenging. For this reason, we developed a technique for direct visualization of calcium deposits on opaque surfaces based on ARS fluorescence properties.

So far, ARS fluorescent properties have been exploited in the context of bone development and regeneration studies [21], often being used to visualize mineralized tissues in vivo [6,19]. However, ARS fluorescence has been used for a qualitative morphological visualization of matrix mineralization. Conversely, ARS staining quantification, as an expression of the extend of the osteogenic process, is usually obtained by measuring the optical absorbance, after the ARS-calcium complex formation. Our data showed that when taking into account the 2D-fluorescence spectra, it is possible to obtain an optical fingerprint of ARS-calcium formation by visualizing a saddle point in the 2D spectra. Indeed, we showed that not only the ARS optical density and the fluorescence linearly increase with concentration, but also that the formation of the spectra saddle point is strongly related to the ARS-calcium complex. This structural fingerprint can be used to provide a real-time read out of calcium complex formation during matrix mineralization, both in vitro and in vivo. We compared the classical quantitative osteogenic quantification based on the OD and the fluorescence 2D-spectra, both in situ and after extraction. These four methods gave similar results with error <10%. The 2D in situ fluorescence spectra were also acquired on GO, an opaque absorbing material and hence, it is not suitable for in situ OD measurement. The fluorescence signal allowed the detection of the bone matrix distribution over the entire GO surface. With this method, we characterized the GO influence on the osteogenesis process both alone and after osteo-inductive treatment. Space-filling scaffolds/coatings allow bone ingrowth and regeneration. Provision of a suitable 3D structure is important to obtain good implant incorporation through rapid vascularization [22]. Further uniform formation of the bone matrix is crucial [23], but the opaque materials allow quantification of matrix deposition only after labelling with ARS and extraction from the surface or after fixation and sectioning of samples. This method will allow a real-time topographical analysis of cell matrix distribution on opaque materials with high sensitivity. Further, the study of mineralized structures after implanting in vivo is also based on the analysis of fixed samples [24]. For live imaging, bone development can be tracked with radiographs in large specimens or with ARS for studying qualitative matrix formation. This technique will improve in vivo visualization based on ARS by offering a high-resolution quantification, unbiased by autofluorescence. This method potentially paves the way to a range of possible exploitations in bone tissue engineering, from in vivo quantification to in vitro bone matrix topographical distribution on opaque materials.

## 4. Materials and Methods

### 4.1. Cell Cultures

All reagents were purchased from Euroclone (Pero, Italy). CMSCs were isolated in primary culture from calvarial specimens, forming a pooled cell culture obtained from unaffected individuals. Cells were characterized as previously described [25]. Our study protocol was designed according to the European Good Clinical Practice guidelines and with the current revision of the Declaration of Helsinki, and was approved by the Ethical Committee of the Università Cattolica del Sacro Cuore, School of Medicine (protocols number A/606/CE/2010 and 19056/14, 9 October 2014). CMSCs at the 4th culture passage were seeded at a seeding density of 2.5 × 10^4^ cells/well, on either “naked” glass slides or graphene oxide monolayer-coated glass slides. To obtain GO-coated glass slides, commercial GO (Graphenea Inc., Cambridge, MA, USA) at 4 mg/mL was diluted with ultrapure water to a final concentration of 1 mg/mL and drop casted on glass slides (120 µL on 12 mm diameter glass slide). The samples were left to dry at room temperature in a sterile hood in the dark and then used for cell cultures. Growth medium (Dulbecco’s modified Eagle medium (DMEM) with high-glucose (4.5 g/L), supplemented with l-glutamine and 1% antibiotics (penicillin 100 IU/mL, streptomycin 100 mg/mL and 10% Fetal Bovine Serum) was changed every two days until the cells reached confluence in culture. Confluent cells were then induced towards osteogenic differentiation using a standard osteo-inductive treatment with the OM (low glucose DMEM supplemented with 10% FBS, 10 mM α-glycerophosphate, 0.1 μM dexamethasone, and 50 μM ascorbate) as previously reported [26]. Slides with cells cultured in standard growth medium and empty slides (i.e., without cells) were used as differentiation and negative controls, respectively. Cells were fixed in paraformaldehyde (4%) for 15 min at room temperature for 7 and 14 days after the initiation of the osteo-inductive treatment, then stained with ARS as reported before [27,28]. Briefly after fixation, 1mL of 40mM ARS was added onto the cell layer and incubated at room temperature for 30 min. After incubation, excess dye was removed and the cells were washed with ddH_2_O. Stained monolayers were visualized by optical microscopy. The Alizarin was then extracted from each sample by dissolving the samples in 10% acetic acid as reported previously [3].

### 4.2. Spectroscopy and Microscopy Measurements

ARS fluorescence and absorption spectra were acquired by means of a Cytation 3 Multiwell reader (Biotek, Winooski, VT, USA). Experiments were independently repeated three times, using three biological replicates and averaging three spectral readings each time. The calibration curve of ARS was obtained after serial dilution of ARS (from a concentration of 0.00125 up to 4 mM). Imaging was performed using Cytation 3 with a 4X objective and exciting with a LED source from 400 to 580 nm (with steps of 10 nm) and using an emission range between 400 and 700 nm. Image analysis and background subtraction was performed as reported before, using ImageJ software [29,30]. Scanning Electron Microscopy (SEM) was performed to evaluate bone formation. Samples were fixed, dehydrated and sputter coated as reported before and imaged with an SEM Supra 25 (Zeiss, Germany) [31]. Imaging of the ARS staining on GO was obtained using an inverted confocal microscope (DMIRE2, Leica Microsystems, Wetzlar, Germany), exciting at 458 nm and revealing between 600 and 650 nm.

## Abbreviations

ARS	Alizarin Red S
CMSC	Calvarial-derived mesenchimal stem cells
DMEM	Dulbecco’s modified Eagle medium
EXC	Excitation
GO	Graphene oxide
OD	Optical density
OM	Osteogenic medium
SEM	Scanning electron microscopy
